# A Swiss Health Care Professionals’ Perspective on the Meaning of Interprofessional Collaboration in Health Care of People with MS—A Focus Group Study

**DOI:** 10.3390/ijerph18126537

**Published:** 2021-06-17

**Authors:** Fabienne Schmid, Slavko Rogan, Andrea Glässel

**Affiliations:** 1 Department of Health Professions, Division of Physiotherapy Bern, Bern University of Applied Sciences, 3008 Bern, Switzerland; avril_schmid@hotmail.com (F.S.); slavko.rogan@bfh.ch (S.R.); 2 Faculty of Physical Education and Physiotherapy, Vrije Universiteit Brussel, 1050 Brussel, Belgium; 3 Institute of Biomedical Ethics and Medical History, University Zurich, Winterthurer Strasse 30, CH-8006 Zurich, Switzerland; 4 Institute of Health Sciences, Zurich University of Applied Sciences, Katharina-Sulzer-Platz 9, CH-8401 Winterthur, Switzerland

**Keywords:** multiple sclerosis, health care professionals, qualitative research, focus groups, narration, interprofessional collaboration

## Abstract

Multiple sclerosis (MS) is a chronic, inflammatory autoimmune disease of the central nervous system mainly of adults ranging from 20 to 45 years of age. The risk of developing MS is 50% higher in women than in men. Most people with MS (PwMS) experience a spectrum of symptoms such as spasticity, continence dysfunctions, fatigue, or neurobehavioral manifestations. Due to the complexity of MS and the variety of patient-centered needs, a comprehensive approach of interprofessional collaboration (IPC) of multiple health care professionals (HCP) is necessary. The aim of this qualitative study was to explore the meaning of IPC in the comprehensive care of PwMS from a HCP perspective. Focus groups (FG) with HCP were conducted, recorded, and transcribed verbatim. The sample contained HCP from three MS clinics in different phases of care and rehabilitation. Four main categories emerged: (a) experience with IPC, (b) relevant aspects for IPC in patients’ treatment, (c) differences in in- and outpatient settings, and (d) influence of patient perspective. IPC plays a crucial role in HCP perspective when treating PwMS, which can benefit from an IPC therapeutic approach because HCP work together in a patient-centered way. The inpatient setting of HCP strongly supports the implementation of IPC. This prerequisite does not exist in outpatient settings.

## 1. Introduction

**Figure 1:**
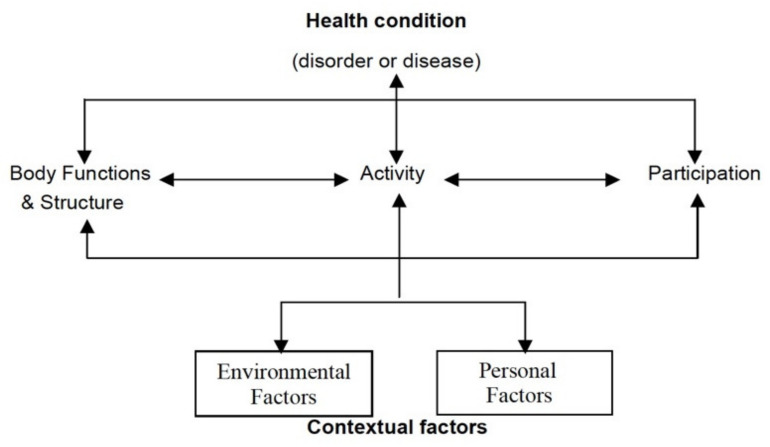
International Classification of Functioning, Disability, and Health (ICF) model of the World Health Organization [5].

Worldwide, 2.5 million people have been diagnosed with MS [1]. In Switzerland around 15,000 people are affected, mainly adults between 20 and 45 years. The risk of developing MS is 50% higher in women than in men [2]. The diagnosis of MS changes a life fundamentally and often requires a restructuring of life. MS is a chronic disease that represents an irreversible presence of disease conditions or damage of the nervous system. In order to promote self-sufficiency, maintain functional ability and prevent further disabilities, the entire environment of the person is rearranged [3] and people-centered care is needed. Rehabilitation is a people-centered health strategy with a set of interventions designed to optimize functioning and reduce disability of patients. The World Health Organization (WHO) integrated people-centered care concept places humans rather than their disabilities and diseases at the center of health care delivery [4]. To better explain the spectrum of limited disability and functioning of people with MS (PwMS), the help of the biopsychosocial model is used. The biopsychosocial model (Figure 1) forms the basis for International Classification of Functioning, Disability, and Health (ICF) [5]. ICF was designed as a common language and data standard that can be used for multiple purposes and in different environments [6].

MS, a chronic disease, is often called “the disease of a thousand faces” [7], which underlines the complexity of MS and the broad variety of patient-centered needs which addresses all dimensions of the ICF model. To achieve comprehensive health care management of PwMS, an approach of interprofessional collaboration (IPC) and comprehensive health care management is necessary. To provide comprehensive health care management from the patient perspective, individual preferences, requirements, and moral concepts which ensure that the patient guides all health decisions should be included. The patient’s perspective and associated quality of life serves as a guide for designing further procedures and MS treatment strategies.

Successful patient-centered management of a complex neurological disease such as MS requires ongoing interactive relationships between the patient and multiple health professionals to take care of different needs. As it is unusual for one profession to deliver a complete program of care in isolation, high-quality care depends upon professions working together in interprofessional teams [8] (p. 1). There is an increasing interest in the ability of health care professionals (HCP) to work together, and in understanding how such collaborative practice contributes to primary health care. The WHO [9] (p. 4) defines IPC practice as “multiple health workers from different professional backgrounds working together with patients, families, caregivers, and communities to deliver the highest quality of care”. Due to the multifaceted and complex nature of MS, an IPC approach is necessary to offer high-quality patient-centered care. Physiotherapists (PT), speech and language therapists (SLT), and occupational therapists (OT) play a key role in the comprehensive MS therapy team. Qualitative studies have explored the perspective of PwMS and their views on IPC access and, in general, their view on MS [10,11,12,13,14]. In general, studies which discuss health care in its pure form focus on competencies, implications, roles, education, and costs but do not specifically address different diagnoses such as of MS [15,16]. The few studies concentrating on the health care professionals working with PwMS address the following topics: needs of patients, and health care professionals’ beliefs, treatments, and education [17,18,19,20]. These studies investigate the views and experiences of different health care professionals who work with modified treatments or exercise interventions for PwMS.

The aim of this study was to explore the perspective of specific health care professionals and their perception of IPC in comprehensive health care of PwMS. It will be specified by the following research questions:

(a) What is the perspective of health care professionals regarding the meaning of IPC in comprehensive health care of PwMS?
(b) How does experience with PwMS affect the health care professional’s IPC?

## 2. Materials and Methods

To answer the research questions from the perspective of specific health care professionals and their perception of IPC in comprehensive health care of PwMS, we decided on a qualitative methodology by using focus groups in an outpatient setting.

### 2.1. Study Design

The reporting of this study was in accordance with consolidated criteria for reporting qualitative research (COREQ) [21]. To answer the research questions, a qualitative study based on a phenomenological approach and including focus groups was conducted between August and September 2018 with health care professional staff from three MS clinics specializing in different phases of care and rehabilitation, in three cantons, in the German speaking part of Switzerland. The methodology explored experiences and attitudes of research participants and depicted objects from the perspective of how they are experienced, thus allowing a search for the essence of the phenomenon [22]. Life conditions become relevant for the world when described by subjective meanings, which can be recorded in qualitative studies [23]. Qualitative research has an openness to the field of research, which enables knowledge gaps to be closed and new fields to be opened.

A characteristic of qualitative research is the studied reflexivity of the researcher’s actions and perceptions in the field, understood as an essential part of the knowledge and not as a source of disturbance to be controlled. As physiotherapists, the authors of this study have a knowledge background as health care professionals but not as physiotherapy specialists in the treatment of PwMS, in contrast to the interviewed health care professionals. Nevertheless, the authors’ own experience in relation to IPC in the inpatient as well as in the outpatient setting naturally brings with it a prior understanding on the part of the researchers. In qualitative research, the subjective perception of the researcher as a component of knowledge is used. The cognitive principle of qualitative research is the understanding of complex contexts rather than explanation by isolating an individual relationship [23].

In order to obtain some broader insights into the perspective of health care professionals’ experiences, focus groups were conducted. This approach encouraged participants to consider the views and opinions of other participants because it was presented in a more natural environment than that of an individual interview, because participants influenced each other just as they do in their daily life [24] (p. 7).

The current study was approved by the cantonal Ethics Committee of Zurich, Switzerland (BASEC Nr.: Req-2018-00440, 14 June 2018). An approval by the ethics committee was not required because the project does not fall within the scope of the Human Research Act (HRA). A declaration of non-responsibility for this study was obtained. The study was performed in accordance with the Declaration of Helsinki [25]. All participants signed written informed consent. Personal data of participants were anonymized in all transcripts.

### 2.2. Sampling and Participants

To recruit health care professionals from in- and outpatient settings in the German-speaking part of Switzerland, different strategies to contact potential participants were applied: we acquired an open-access contact list of MS associations Switzerland, and contacted health care professional associations specializing in MS, specialized clinics for PwMS, and looked at specific outpatient settings for PwMS. Krueger [26] (p. 83) states that the best strategies for finding participants are existing lists of people fitting at least some of the predefined criteria. The goal was to have at least one person per focus group from each profession (PT, SLT, OT). Potential participants were contacted by mail or phone. A purposive sampling selection of the most productive sample was used to answer the research questions. Physiotherapists, speech and language therapists, and occupational therapists aim to enhance the daily living for persons with and without health restrictions through specific interventions which have an impact on a biological, psychosocial, and social levels. All three professions integrate both the material and the social environments of their clients in this transformation process [27] by supporting cognitive, sensory, and motor development. While other health care professionals, such as doctors, neuropsychologists and nurses, play an important role for PwMS, these professions are entrusted with other tasks.

### 2.3. Data Collection

To capture health care professionals’ experiences, a semi-structured interview guide with open questions was developed to give participants the opportunity to describe and deepen their opinions more flexibly, led by topics. In contrast to standardized questionnaires and interviews, with this approach the moderator has the option to inquire about and clarify unclear statements [28] and a specific dynamic group interaction is created [26]. The semi-structured interview guide for focus groups was pre-tested and was based on a literature search to contextualize the theme of IPC with PwMS and contained the lead questions listed in Table 1. Questions were asked in positive and negative ways, e.g., supportive factors, chances and barriers, directions. Each focus group was led by two researchers and a moderator (AG/FS); furthermore, one researcher (FS/AG) took notes. For sociodemographic data, participants filled in a questionnaire.

### 2.4. Data Analysis

Focus groups discussions were in Swiss German (Swiss dialect), audio-recorded and transcribed verbatim into High German based on rules by Dresing and Pehl [29] by using the software program F5 (2004–2007—version 2.0.2). All focus groups transcripts were coded by using the software MAXQDA program (10.0 Standard 2018/04) based on the six phases of Braun and Clarke’s thematic analysis [30,31]. The aim of qualitative thematic analysis is to process communication material, which was performed by an inductive and deductive categorization (Table 2).

### 2.5. Quality Assurance

For data quality assurance, the COREQ checklist was applied [21] and criteria of Flick, Kardorff, and Steinke [23] were considered: data security regulations regarding data storage were observed throughout the study. For peer-review, a pre-test for obtaining feedback on the focus group questions in the topic guide was conducted twice. Multiple strategies to capture the data were applied [26] (p. 145). Beside audio-recording and the minutes during all focus groups, field notes were collected by a second researcher. After each focus group, both researchers reflected on the process for potential improvements by written debriefing. Before starting data analysis, both researchers conducted training and drafted a coding scheme based on the questions. To ensure trustworthiness of data, the accuracy of the transcriptions was reviewed including application of rules and writing memos. For peer review, a second researcher coded 25% of data of the transcripts. Both researchers discussed the coding results for common agreement on each concept. For member checking, all participants were offered the transcripts. A plain language summery of the results was sent via email.

## 3. Results

### 3.1. Sampling and Participants

Three focus groups were conducted with 13 health care professionals represented by six PTs, four OTs, and three SLTs in three German-speaking neuro-rehabilitation centers from July 2018 to April 2019. None of the participants dropped out. The average duration of the focus groups was 53 min, with a range from 33 to 77. Contacts to clinics resulted from a therapist list of the Swiss MS Society and from directly writing to leading clinic specialists. Answers were obtained from inpatient and semi-inpatient institutions. With a total of three persons, i.e., one person per focus group, SLTs were slightly underrepresented. Sociodemographic data of focus group participants are presented in Table 3.

All participating health care professionals had MS specific training; most frequently mentioned (7/13) were being trained in the Bobath concept or having obtained a Swiss Certificate of Advanced Studies (CAS) in MS (3/13). Regarding specific training in IPC, only three out of 13 participants had undergone external training. Internal training on IPC takes place in all visited clinics. The health care professionals were not evaluated individually according to their profession. As [26] states, caution must be exercised when focus group participants represent different categories of people as is the case in this study. If opinions of a single category of people are to be determined, a focus group with that specific category of people is needed. Krueger further testifies that a focus group of diverse people is not sensitive enough to discover trends of subcategories of people.

### 3.2. Data Analysis

**Figure 2:**
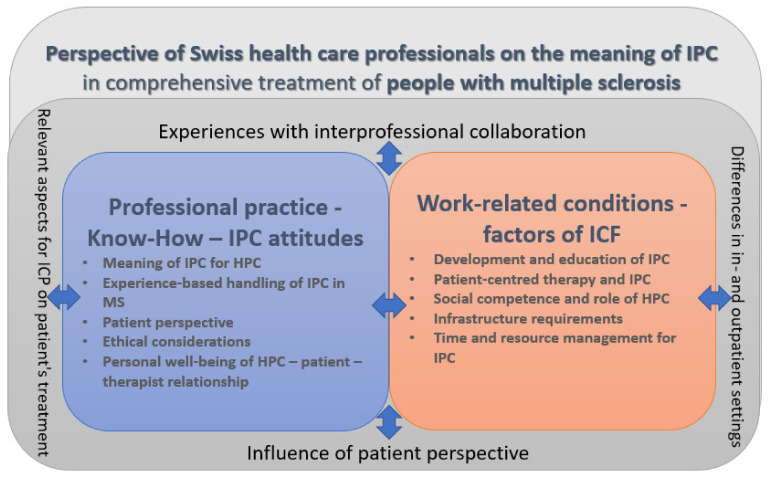
Overview of results from a health professional perspective (health care professionals = HCP).

The codes that were generated were grouped into themes and categories. The following four main categories emerged from the data out of three focus groups: (a) experience with IPC, (b) relevant aspects for IPC on patients’ treatment, (c) differences in in- and outpatient settings and (d) influence of the patient perspective (Figure 2). Sub-categories are described as follows, including sections and examples of health care professionals’ quotations (see also Supplementary Figures S1 and S2).

#### 3.2.1. Professional Practice—Know-How—IPC Attitudes

##### Meaning of IPC for Health Care Professionals

This section describes health care professionals’ statements about their individual definitions, understanding, and relevance of IPC in managing a comprehensive health care situation of PwMS. Collaborative agreements between individual professions facilitate the achievement of goals.

*«For me, interprofessional means pulling together on the same rope and if possible, being able to do inpatient work on the same goals in a discipline-specific way (--) and not sinking into parallel worlds where everyone works in a different direction». (FG03; 118)*

*«But the goal or the patient should actually be in the center. And the goal should be defined as realistically as possible. And, of course, we also need to talk to each other. But that’s what I understand by the definition of interprofessional cooperation». (FG01; 114)*

Participants discussed IPC as a huge benefit both for the patient and for the health care professionals themselves to work together for and follow the same goals. IPC gives the health care professionals a holistic picture of the patient based on ICF. By bringing together a large number of health care professionals with a highly specific training in MS, not only can the patient benefit but also the therapist. IPC also means an accumulation of knowledge and experience in one place.

*«You also know who works a lot with PwMS, who can I ask, if this is not my subject. For example, if someone has breathing problems, I can ask this therapist». (FG01; 94)*

*«But we also have a holistic picture of our patients, it is not simply enough just to check out our own professional area. We are also always in contact with the physiotherapy, how far they can go. Because this also has a direct impact on patients’ leisure time and independence and daily routine». (FG02; 21)*

##### Experience and Handling of IPC in MS

Over the years in specific health care of PwMS, the participants were able to experience and learn a great deal about IPC. The exchange among the health care professionals gives them insights not only into their own specialist area but also into that of the other professionals involved. By forming mixed discussion groups, the risk of focusing too much on one’s own profession-specific goal and thus possibly losing the overarching goal can be reduced.

*«I think it makes it a lot easier. IPC is important to look and see, maybe I have forgotten something. There are cognitive problems for example, and there are also other problems. And that’s where I see the importance of working together in addition to my own work». (FG03; 120)*

Participants learn to see the treatment of PwMS in a broader and more comprehensive context as many people are involved. IPC is described by the health care professionals as a process to be learnt which one needs to grow into. As an employee, people will also learn the employer’s philosophy regarding IPC and the corresponding approach to IPC. At best, you will learn to identify yourself with this approach of the house. The health care professionals experience how all the other professions contribute towards reaching the patient’s goal.

*«That you can only really work at its optimum if you work interprofessionally. And this fundamental attitude that I believe you have to learn to live». (FG01; 47).*

Conducting a conversation with a person who has a chronic disease is complex. Participants said that experience with PwMS has shown that especially if you have got to know each other so well over the years due to this chronic illness, it is essential to take a clear stand and to be honest with patients. It is considered important to apply a common communication as an interprofessional team.

*«You have to be clear about what you say, so that the patients understand. That they can understand when you do a lot of therapy for three weeks, but no progress shows up». (FG02; 54)*

*«Then you quickly reach limits of what you can do in the conversation with the patient, for example, how can I tell him that now? These are special challenges. So, the topic of conversation somehow, how can I justify myself? ». (FG01; 74)*

##### Patient Perspective

The patient perspective plays a crucial role in health care and quality assurance. During a disease episode, patients get in contact with all therapists involved in care on an in- and/or outpatient basis. They can review the entire care chain. During their therapies, health care professionals perceive or provoke emotions from patients. They get insights into the patients’ personal views, experiences, and life stories. The patient also enters the therapy with a form of expectation regarding, for example, the quality or type of therapy (treatment approach). The PwMS experience contact with other patients in the specialized clinics who are diagnosed with the same illness but possibly being at a different stage. This is perceived by therapists as both an advantage and a disadvantage.

*«Yes, patients see their own future». (FG02; 94) «That is another condition to live clearly in the context I am getting worse and worse, I have a chronic illness, what awaits me, even young people with family». (FG01;67)*

*«For example, during the first stay, if the therapist recommends a foot lifter splint, they say no and generally reject everything. And over time, during the second or third stay, they see that other PwMS are better off with the same aids and then they accept it. They profit very much from each other». (FG02;71)*

As a therapist, one must learn to understand the patient’s point of view and to be able to take their position. On their own initiative, PwMS often try to get the best out of themselves or to improve or maintain their quality of life as well as possible. Patients develop coping skills; thus, the health care professionals mentioned coping strategies for everyday actions.

*«For example, we have a patient who drives her wheelchair with a self-made mouth stick when her hand gets tired». (FG02; 66)*

*«Yes, they have surprising coping skills, also to some extent self-destructive. How they deal with their problems». (FG02; 62)*

*«And there they come up with their own ideas and you reflect and think together. You can’t get such knowledge out of textbooks and reference books. For me, that has actually a lot of value as a therapist». (FG02; 66)*

##### Ethical Considerations

The four biomedical principles of Beauchamp and Childress (2001) [32], which are (a) respect of autonomy, (b) justice, (c) beneficence, and (d) non-maleficence, are an established, widespread framework applied in challenging ethical questions of health care. When working with PwMS, health care professionals can sometimes find themselves in difficult ethical situations and are required to make adequate decisions, especially in difficult cases when two principles seem to be conflicting or even exclude each other. In MS changes in cognitive abilities are not infrequent, limiting patients in making autonomous and self-determined decisions. Therapists are faced with ethical challenges because the questioning of important topics can often not be made by explicitly asking the patient. When does it make sense to decide something for the patient that takes away a part of his or her quality of life, but is no longer acceptable for safety reasons? Balancing damage and benefit put health care professionals and PwMS in a conflict situation.

*«And with PwMS, it’s often the case that you don’t want to take anything away from them. So, you know, it’s probably already dangerous if he eats the peanuts now. But he loves them more than anything else and that would be a big incision for him now, if I said that he shouldn’t eat them anymore. And then what does this neuropsychological clarification mean? That would mean in three quarters of the cases that they can no longer drive». (FG01; 71-83)*

From the therapist’s point of view, there is a need to be able to assess the change in individuals due to MS because there is a risk that cognitive limitations of PwMS can also be misinterpreted as patient reluctance or lack of motivation. The principle of autonomy often conflicts with beneficence. Health care professionals do not want to take anything from the patient that limits his or her autonomy but at the same time they want the best for him or her.

*«...but if you are not that aware of the cognitive limitations, you often ask the patient and think the patient does not want that. But often the patient can’t really estimate». (FG01; 79)*

##### Personal Well-Being of Health Care Professionals/Patient–Therapist Relationship

Health care professionals reported that at some points they reached their personal limits in dealing with PwMS. What makes the therapeutic work successful? It is not only about well-founded training and the professional approach. Personal commitment and engagement are key requirements for working in an IPC environment. For successful treatment, the health care professionals have to take an individual approach tailored to the personality and peculiarities of this specific PwMS. This means being touched by the fate of this person, adding ideas, developing an image of this person and getting emotionally involved. It is an intense and close relationship. This patient–therapist relationship offers possibilities for disagreement and points of conflict.

*«And then you feel very stupid. And I like to think I’m over 30 years old and he insults me like a little girl. I know how to react to it. I also see him as a patient who has a terrible illness, who can’t deal with it. His illness changes him, we just treat him, but yes. Maybe he will be nice someday». (FG02; 80)*

*«I am overwhelmed when a patient approaches me very negatively or very impudently. That is difficult for me. There I am.... I already have a few strategies, but if all this is useless to me, if I am only loaded, then I know that my therapy has absolutely no effect at that moment. And then I also take the liberty to stop it. Yes, I have to explain that to my colleagues afterwards». (FG02; 77)*

#### 3.2.2. Work-Related Conditions—Factors of IPC

##### Development and Education of IPC

IPC has undergone a big change in recent years. Most rehabilitation clinics work interprofessionally and have developed and implemented organizational structures to offer IPC educational concepts. All participants explained they have gained more and more insight into the other professions, demonstrated by the fact that one professional group knows MS-specific instruments from another group of health care professionals.

*«I can really see that in MS these are the pioneers of interprofessional cooperation». (FG01; 25)*

*«It has improved more and more over the last 10 to 15 years. It already starts with recording reports. All professions are present together and they are checking among each other what is the focus of this clinic stay. The therapy package will be put together accordingly. In the last two, three years, collaborations have become better, since we have a common team room.... On the other hand, when aids are needed or when positioning is required for the more severely affected patients, intensive collaboration is required. Or you make splints and we do the positioning together». (FG02; 18)*

*«For me, insights into job descriptions of the individual professionals are important. Because in recent years we have been able to gain insights from time to time. For example, we had a detailed explanation of how to swallow and how important that is. And if I have a good insight, I can put myself better into it. We had gotten further training; and mutually I know more about occupational therapy and speech therapy than if I didn’t work here. (FG02; 38)*

##### Patient-Centered Therapy in IPC

This section presents statements about the patient being the center of therapy and decision-making. IPC is a collaborative exchange that is carried out for the patients’ benefit. A key statement of the health care professionals was to focus on the patient’s view or him- or herself as a human being.

*«But I think the goal should be that the patient’s view of what he wants always comes first». That’s how you should put it. And then perhaps a therapeutic goal can result from it». (FG01; 123) (FG01; 123)*

*«The fact that the patient is also a component of this interprofessional team includes him as a partner». (FG01; 106)*

Additionally, challenges were mentioned. Health care professionals try to respond to wishes and specific needs of individual patients as far as possible.

*«But it’s just like doing a balancing act to say that is what the patient wants to do, but that is what I want to work towards now, something different». (FG01; 128)*

*«These people also usually come into therapy with a very precise idea of what to do now, what the next step could be and what they need. And picking them up, what they imagine and at the same time being able to bring in the therapeutic expertise is a bit of a balancing act». (FG01;64)*

##### Social Competence and the Role of the Health Care Professionals

In the focus groups, participants discussed in depth the influence of the health care professional’s own personality and social competences. The personal background (beliefs, education), motivation, mood, empathy, and perception of situations were all mentioned as factors that can have a positive and negative impact on IPC. IPC also means a division of labor, making joint cross-disciplinary agreements, having good communication between each other and knowing one’s own role and importance in this interprofessional health care team.

*«The one who sees a need addresses it». (FG03; 41) and «Communication is everything». (FG02; 107)*

*«...Should I do it now or will the SLTs do it? Regarding aids with communication devices, who does what? So simply that it does not run twice or that it will not get forgotten. There are both possibilities here». (FG01; 49).*

Health care professionals stated that they have to be able to step back within the team and also to possess the ability to perceive when their role is needed and when not. It takes a high degree of reflexivity to work in this environment in order to be, and continue to be, able to cooperate. At the same time, a certain instinct is required to know when to resign and to commit oneself to represent the patient’s goals in one’s own therapy.

*«To make arrangements and to be able to withdraw. If necessary, to be able to withdraw and remember that I may not be the main person (therapeutic discipline) who has to contribute something». (FG01; 155)*

*« I also think that one can ask easily and admit oneself, when I need help». (FG01; 94)*

Connected to social competence, expressions such as emancipation, hierarchical structures, communication at head level, and rivalry were mentioned. IPC means to see the holistic picture of the patient and to look beyond one’s own professional area.

*«That you can also say, yes I step back now because the patient does not want my therapy. That you are also flexible with structures and hierarchies. That you have a level where you can really communicate with your team». (FG01; 118)*

*«I think this is very important, the emancipation from these old structures where the doctor was the leader who always knew everything. Which may be partly true, but often not, because it’s actually not possible to know everything in such complex settings». (FG01; 119)*

The personality of the therapist regarding the interest in implementing IPC is a basic condition. IPC means being able to work in a team and being interested in areas other than one’s own. The fact that someone who works in the health care sector already has a high degree of social competence is a good prerequisite. Identification with IPC is an important factor for working successfully and for long-term satisfaction.

*«I think similarly, one must have an interest. I wonder what patients do in speech therapy, what their cognitive abilities are and what they do in physiotherapy. I need to have an interest. It always takes self-initiative» (FG02; 42)*

*«...and we read each other’s reports or then just go and ask or discuss when we have a concern». (FG03; 30)*

##### Infrastructure Requirements

An often-cited topic was conditions or infrastructure of the employer. Clinic internal online networks provide health care professionals with information concerning treatments of all professions working around with a patient. Additionally, sharing treatment or break rooms among different health care professionals can stimulate IPC.

*«What makes it easier is our process in the “Phoenix” system. We have made it very professional and now we have a sheet where all disciplines note their current state of therapy». (FG02; 92)*

*«Our infrastructure makes it easier to reach our destinations. Plus, that new ideas are implemented relatively quickly here. We therapists work door-to-door with each other, there is a lot of exchange». (FG02; 94)*

Larger centers have a local advantage; all disciplines are combined in one place. This makes it more efficient for having conversations in specific time slots for meetings or in the corridor. Spontaneous meetings of two disciplines were especially mentioned as supportive and productive in relation to keeping on track to reach patient-oriented treatment goals.

*«Or when we walk through a therapy room and see the colleagues from the physiotherapy treating the patient right now and we have a few minutes, then we observe briefly or can then use time for a brief exchange». (FG02; 22)*

Health care professionals also mentioned that in controlled settings, as is the case in inpatient care, IPC is clearly easier to access compared to outpatient settings. Costs (time is money) for exchange, conversations, and documentation with other health care professionals regarding patient-specific needs for therapists who are working in strictly outpatient centers are higher and financially more difficult to bear. In the outpatient area, for example, only reports for accident insurance, disability insurance, and military insurance can be billed, and this only if the insurance company explicitly requested a report.

*«Because the case with us in the outpatient clinic is that I have to consider whether I can find time for a phone call. I cannot charge this time as physiotherapy. And a lot of things that you normally wanted to contribute to quality just will be dropped and left away». (FG01; 110)*

*«You might be a bit more in your chamber and working on your own thing if you do it on external outpatient care. It rarely happens that I consult the responsible doctor for an outpatient patient». (FG03; 137; FG03; 158)*

##### Time and Resource Management for IPC

Structured time frames for interprofessional patient meetings were emphasized to be important. Meetings are held at regular intervals and are a great facilitation to maintain IPC. Through shared discussion time, individual perspectives of disciplines on problems and goals can be reflected. This applies primarily to inpatient and semi-inpatient facilities but not to strictly outpatient settings. IPC needs a lot of time, thus also causing costs, which is seen as a risk for developing IPC.

*«Yes, and I think the big difference for me is that there are also structures provided for this exchange in the inpatient sector». (FG03; 139)*

*«In the day clinic they take place every week, but each time different patients are discussed. Then one has 10 to 20 min time, where one discusses the patient with the interprofessional goals, agrees on these and names the main problems». (FG01; 45)*

## 4. Discussion

This current study focused on exploration of the Swiss health care professionals’ perspectives regarding the meaning of IPC in comprehensive health care of PwMS and what influence the experience with PwMS has on IPC of the health care professionals. This study highlighted that a patient-centered therapy in IPC means that PwMS are placed in the middle and their needs and demands are incorporated into the therapy and structure it. The importance of the patient perspective in the rehabilitation process manifests itself and corroborates the findings of other studies. Heesen et al. [33] concluded that 80% of PwMS require an autonomous role in treatment decisions. Moreover, Tractenberg et al. [34] concluded that there are qualitative differences in therapy outcomes that are using a patient-centered approach. Cutler et al. [35] summed up that including the patient in the health care professionals team makes sense for long-lasting treatments such as physiotherapy or mental health services but less for acute treatment like emergency care.

However, the ideas of PwMS and health care professionals can also diverge. The study of Rothwell et al. [36] could already show that the understanding of disability in MS differs between doctors and PwMS. So identifying needs and demands of PwMS is crucial for a comprehensive IPC. This highlights the aspect that the meaning of IPC for a health care professional can also be understood in different ways. As demonstrated in patient perspectives in this study, patients are increasingly searching the internet for information. With a diagnosis of a lifelong disease, PwMS inform themselves in depth about their symptoms and disease progression.

The findings in the section on personal well-being of health care professionals and the patient–therapist relationship in this study demonstrated that personal commitment and engagement are always required. However, there are situations as a health care professional treating a patient with MS in which one reaches personal limits and even has to stop his or her therapy. In such cases it is important for therapists to distance themselves from these high and difficult-to-reach expectations and hopes of PwMS. It challenges therapeutic success when people are denied reaching common defined treatment goals.

For the successful implementation of IPC, certain conditions are needed. Study results about infrastructure, time and resource management requirements are consistent with those in the literature, which states that the use of technology for the implementation of effectual IPC is traced as a core element [37,38]. A lack of compensation for interprofessional collaboration services is considered an influential hindering factor. Interprofessional collaboration among stakeholders has so far been based on the commitment of individuals. Remuneration is provided in the outpatient setting for physicians, but not for health care professionals [39]. Further elements, such as the geography of workplace and schedule, can have an influence on IPC. There is a need for tools to share and support face-to-face communication with institutions or teams [8]. The literature states that hierarchies can suppress IPC [40]. This can also be found in the results concerning the social competence and role of health care professionals. Important key factors are to ensure that every team member understands his or her own role, his or her field of work and to enhance respect for each other within the IPC team [15]. Therefore, it is crucial that members of the IPC team show respect for the competences of other health care professionals within the team and work together to achieve common patient goals. Health care professionals must also be able to admit a lack of knowledge without losing trust of PwMS. In the literature, skills and competences within teams are defined as key requirements for an effective work performance [15].

However, not only do health care professionals have roles, but patients and their engagement are also important within IPC, which is indicated in the category ethical considerations. Engagement of a patient has the potential to increase a health care professional’s awareness that their actions can have real consequences for individuals, which in turn leads to moderate risk-taking behavior [41]. Still, as can be seen in the example of PwMS, effects of a disease can have an impact on patient engagement with health care professionals. Their illness, mental state, ethnicity, language spoken, and mental capacity can significantly affect their ability to engage themselves with the health care professional.

The experience and handling of IPC in the MS work field shows that the neurological disease provokes coping skills from a patient perspective but also in health care professionals dealing with PwMS. Coping was first specified in the 1960s as the effort of cognition and behavior to handle, reduce, or tolerate inside and outside demands which cannot be handled with one’s own available resources [42]. In the literature the term is not only used in connection with positive results. This was also the case with the participants in this study. The health care professionals stated that PwMS partly react with the suppression of the problem. This suppression coping reaction stays in line with typical psychological adjustments among PwMS [43].

The Swiss Federal Office of Public Health (FOPH) also recognized the need for IPC. By means of a support program called “Interprofessionality in Healthcare 2017–2020” they try to improve IPC in the Swiss health care system and increase its efficiency [44]. Based on the program outcomes of interprofessional collaboration in health care, four policy briefs—findings and recommendations for target group-specific implementation (a) interprofessional education, (b) inpatient setting, (c) outpatient setting and (d) psychosomatic setting and interfaces—were published in May 2021 [45]. The findings are intended to be used by persons responsible at a local, cantonal, and national level, by communities, as well as private and public organizations, to promote interprofessional education and practice in the health care system in Switzerland.

### Limitations

For a comprehensive in- and outpatient health care setting for PwMS, different health professions are relevant for IPC based on the need of care and depending on the phase of this life-long disease as physicians, neurologists, psychologists, nurses, pharmacists, podiatrists, and others including therapists. Our study was focusing on IPC in health care professionals; as therapists this means physiotherapists, occupations therapists and speech therapists. By reflection on the results, this will limit this study related to the comprehensive outpatient health care setting for PwMS. The findings of this study cannot answer the question of what the IPC experience in a strictly outpatient setting is, and how its implementation takes place. Although attempts were made to obtain outpatient results, outpatient health care professionals were not willing to make themselves available for focus groups or even individual interviews. The reason consistently given was a lack of time. To minimize the risk of losing the voice of outpatient health care professionals, one strategy was to conduct individual interviews with health care professionals in the outpatient setting before losing this information completely. However, the outpatient health care professionals were not supported to participate in a focus group during their working hours and outside their working hours they were not willing. Furthermore, the creation of outpatient focus groups was made more difficult by the different local workplaces. For future project methods, an online focus group approach could be a useful tool to include hard-to-reach health care professionals to facilitate their participation. All health care professionals who were included worked in inpatient rehabilitative settings and were thus locally limited. The most noticeable aspect when writing to outpatient health care professionals was that it was extremely rare that PT, SLT, and OT worked all together in a strictly outpatient practice. This may indicate further organizational problems for PwMS who depend on all three forms of therapy.

By reflecting the results, we would like to acknowledge that only some of the health care professionals approached for the study agreed to participate. This means we do not know the opinion and attitudes toward IPC of those who declined.

A critical look at this work reveals that the interviews were conducted by a professional colleague and master student. This position is, in fact, an insider position, which can be advantageous, for instance, because both sides share a common language and nomenclature. In a focus group setting, the researcher has several functions such as moderator, listener, observer, and eventually analyst [26]. A disadvantage is that the interviewer’s own experiences, norms, and values regarding the phenomenon to be investigated are involved. In this study, health care professionals working in inpatient, semi-inpatient and rehabilitative settings were interviewed. In contrast the personal perceptions of the author herself, working in a strictly outpatient setting, is that IPC does not exist in the outpatient care of PwMS in Switzerland. However, this observation could only be compared to a limited extent with results from this study.

## 5. Conclusions

The study wanted to examine two research questions. On the one hand, ‘What is the health care professionals’ perspective regarding the meaning of IPC in a comprehensive health care of PwMS’? The findings suggest that IPC plays a crucial role in specific inpatient MS clinics in Switzerland. Moreover, it was revealed that overall PwMS can only benefit from an IPC therapeutic approach because health care professionals work together in a goal-oriented and patient centered way. It could be shown that close location (inpatient setting) of health care professionals strongly supports the implementation of IPC.

On the other hand, the second research question, ‘How does the experience with PwMS affect the health care professional’s IPC?’ could be answered as follows: This is a disadvantage for PwMS in outpatient settings because this possibility does not exist in Switzerland so far. In settings where individual professions work at larger distances, less or no IPC seems to take place. Further research in outpatient settings about the implementation prerequisites of IPC for PwMS and health care professionals’ views is needed. The outpatient sector shows a strong dispersion of the professions, which may also make it difficult for the PwMS if an interprofessional approach should be desired. Incentives for cooperation in the outpatient sector should be created from the field of politics in order to be able to connect health care professionals caring for PwMS in Switzerland.

Health care professionals themselves and health institutions can benefit from these findings to gain an overview of their processes and possible missing contacts for outpatient IPC.

## Figures and Tables

**Table 1:** Questions of focus group interview guide.

Question Order	Questions of Focus Groups Interview Guide
A	**What experiences have you had with interprofessional cooperation (IPC) in the treatment of patients (positive and negative)?** Possible stimulus question: - What do you understand by interprofessional cooperation?
B	**What positive and/or negative aspects do you consider relevant from your perspective as a therapist for interprofessional treatment of PwMS?** Possible stimulus questions: - What is your experience with patients with MS compared to other patients? - What obstacles/challenges can you describe in terms of comprehensive treatment as a therapist in treating patients with MS? - What aspects do you think facilitate/hinder IPC in your work with patients with MS? - What role do you think the patient perspective plays for IPC? - What are the advantages/disadvantages of IPC in your opinion? - What experience do you have with regard to training in IPC?
C	**Do you think there are differences in IPC (knowledge and application) in inpatient and outpatient care?** Possible stimulus questions: - Are there any professional groups with whom more IPC takes place? - What opportunities and challenges can you see for IPC in the provision of health care for people with MS? - What are you personally doing to achieve better IPC? - In your opinion, is IPC demanded and necessary, or superfluous for MS patients?
D	**How do your experiences with PwMS influence the IPC of different health professionals?**
E	**Do you have additional aspects which are relevant from your perspective to add to IPC?**

**Table 2:** Six phases of a thematic analysis of focus groups with health care professionals based on Braun and Clarke (own presentation) [30].

Phases 1–6:	Tasks of Thematic Analysis of Focus Groups
Phase 1: Familiarsing yourself with your data:	At the beginning, all transcripts were read and initial ideas were written down.
Phase 2: Generating initial codes:	Text passages were extracted from all three focus groups and provided with codes that were relevant for the research questions. This coding was inductive, and data controlled. Transcripts of all three groups were independently coded by a second researcher (AG) to improve research rigor. Created codes were compared and discussed by the research team (FS and AG). After coding all three focus groups, one researcher (FS) went through all coded transcripts again for reviewing and competition. Ideas and thoughts relating to every code were written down in memos. After a reviewing evaluation all statements were condensed to one code.
Phase 3: Searching themes:	Codes were collected to identify potential themes. For this, the written memos were used.
Phase 4: Reviewing themes:	The created themes and the associated codes were checked. Memo function was again used to refine definitions of the themes.
Phase 5: Defining and naming themes:	Themes were checked again and then divided into sets based on the questions.
Phase 6: Producing the report:	The individual sets were assigned to categories. A visual representation was carried out of code, topic, and sets.

**Table 3:** Sociodemographic data of participants from the focus groups.

Demographics		Number of Participants
Age	20–30 years 31–40 years 41–50 years 51–60 years No details	2 5 4 1 1
Gender	Male Female	1 12
Profession	Physiotherapy Occupational Therapy Speech and Language Therapy	6 4 3
Work field	Outpatient setting Inpatient setting both	1 5 7
Work experience with PwMS	<1 year 1–2 years 5–10 years 10–20 years >20 years	0 1 7 4 1

## Data Availability

Data are stored at the Institute of Biomedical Ethics and medical History, University of Zurich.

## Supplementary Materials

The following are available online at https://www.mdpi.com/article/10.3390/ijerph18126537/s1, Figure S1: Extract from MAXQDA Coding, Figure S2: MAXQDA Main Sets.
