# Supplementary figures and images for: A Swiss Health Care Professionals’ Perspective on the Meaning of Interprofessional Collaboration in Health Care of People with MS—A Focus Group Study
Source: Int J Environ Res Public Health. 2021 Jun 17;18(12):6537. doi: 10.3390/ijerph18126537 (PMC8297392; doi:10.3390/ijerph18126537)

Figure S2: MAXQDA Main Sets

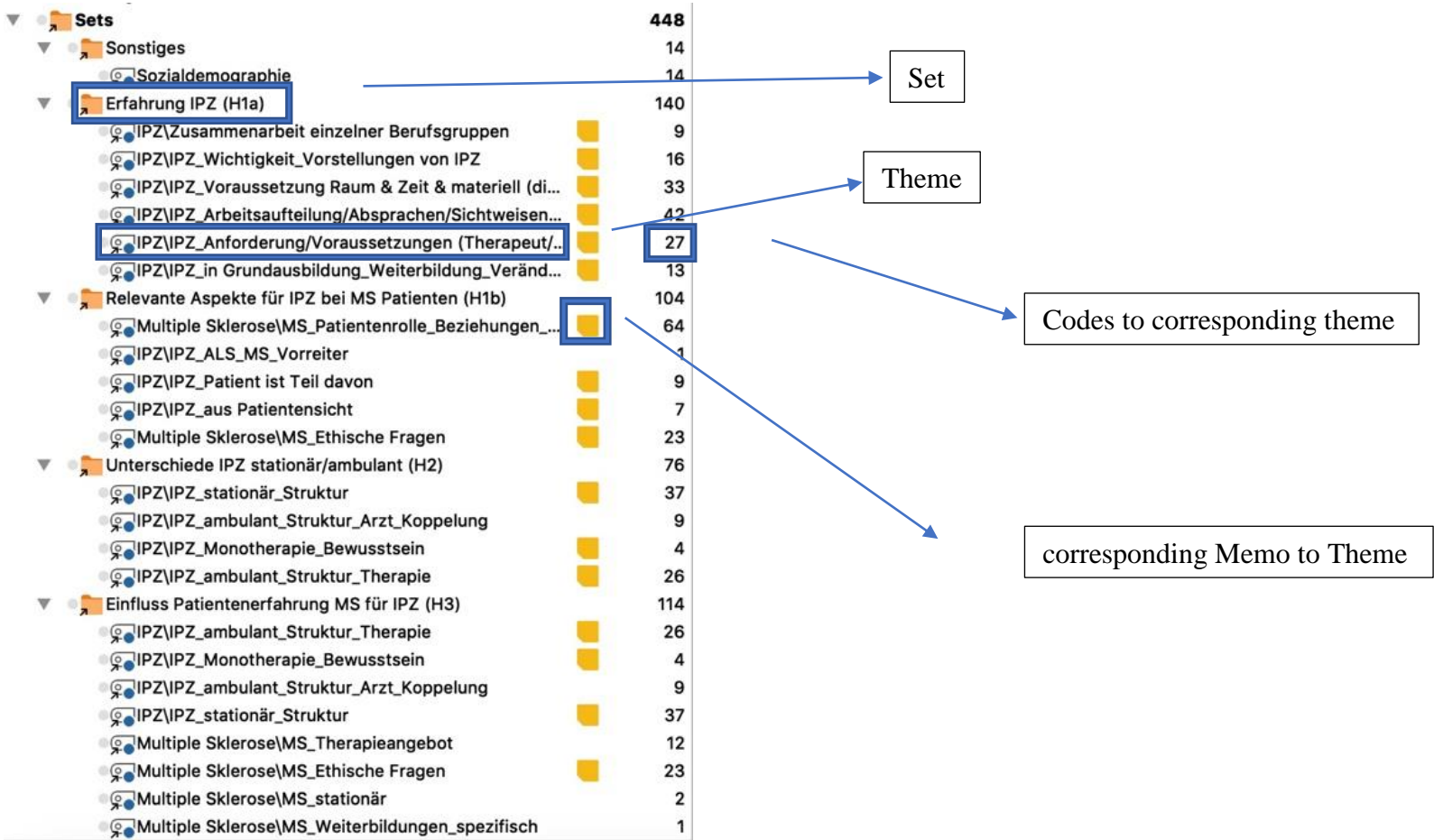

Supplement: Supplementary file 1 [file ijerph-18-06537-s001.zip › s2.pdf]
